# Ethical challenges in residential care facilities during COVID-19: Leaders’ perspective

**DOI:** 10.1177/09697330241255934

**Published:** 2024-06-06

**Authors:** Anna-Carin Karlsson, Anna-Karin Edberg, Malin Sundström, Annica Backman

**Affiliations:** Research Platform for Collaboration for Health, Faculty of Health Science, 4342 Kristianstad University, Kristianstad, Sweden; Research Platform for Collaboration for Health, Faculty of Health Science, 4342 Kristianstad University, Kristianstad, Sweden; Research Platform for Collaboration for Health, Faculty of Health Science, 4342 Kristianstad University, Kristianstad, Sweden; Research Platform for Collaboration for Health, Faculty of Health Science, 4342 Kristianstad University, Kristianstad, Sweden; Department of Nursing, Umeå University, Umeå, Sweden

**Keywords:** COVID-19, ethical challenges, person-centred care, residential care facilities, interviews, leadership

## Abstract

**Background:**

Person-centred care is based on ethical principles, and it is regarded as high-quality care. Care of older persons should embrace person-centredness. During the pandemic, older persons were highlighted as a vulnerable group at risk of developing serious illness and/or suffering death from COVID-19. Several pandemic-related measures were introduced in residential care facilities (RCFs) to reduce this risk, which influenced the possibilities to lead and provide a person-centred care.

**Aim:**

This study’s aim was to explore ethical challenges in relation to person-centredness during the COVID-19 pandemic, from the perspective of leaders in RCFs.

**Research design:**

The study had a qualitative descriptive design.

**Participants and research context:**

Semi-structured interviews were conducted with 26 leaders working in RCFs in Sweden. Data were analysed using conventional content analysis.

**Ethical considerations:**

The study was approved by the Swedish Ethical Review Authority. The participants received oral and written information about the study and gave written consent. The study was conducted in accordance with the Declaration of Helsinki.

**Findings:**

The overarching ethical challenge was *Having to disregard the individual needs of the person in order to protect the group and society*. This included (a) Protecting the group versus promoting the older person’s autonomy; (b) Being forced to lead care based on uncertainty instead of evidence; (c) Striving to provide dignified care but lacking opportunities; and (d) Going far beyond ordinary duty and endangering one’s own and the staff’s health.

**Discussion:**

The ethical challenges meant being torn between the person’s individual needs and protecting the group and society, with clashing ethical principles as a consequence.

**Conclusions:**

The leaders faced ethical situations resulting in undignified and compromised person-centred care, which has implications for stakeholders and management who need to address the work conditions in RCFs.

## Introduction

Like many other countries, Sweden was challenged during the COVID-19 pandemic and the number of deaths increased every day.^1^ Authorities worldwide stated that an early goal was to protect older persons from getting infected with COVID-19.^2^ In residential care facilities (RCFs), several restrictions were introduced to prevent and/or limit the spread of the infection^3^ affecting both residents and staff. The RCF leaders, registered nurses (RNs) and first-line managers (FLMs) had to lead the care under pandemic terms and conditions. This meant that daily care took a back seat, which created ethical challenges, especially in RCFs.

It has been stated that high-quality care should embrace person-centredness,^4^ which has clear connections to the concept of ethics.^5^ Therefore, a hallmark of person-centred care is its ethical foundation.

## Background

Ethical challenges arise in situations where interests and conditions are in conflict, which requires choices and decision-making that affect the outcome of the situation.^6,7^ Preshaw et al.^7^ showed, in a review of ethical challenges in RCFs, that challenges in nursing practice involved clashing principles, that is, respect for autonomy, beneficence, non-maleficence and justice,^8^ as well as a clash with professional responsibilities, confidentiality and duty of care.^7^ Moreover, a major part of the ethical challenges were ascribed to communication and decision-making with care providers, residents and relatives, as well as lack of resources and lacking quality of care provision such as the possibility to provide person-centred care.^7^ Confronting ethical challenges involves a complicated tangle of interests and aspects from different perspectives such as older persons and staff.

The risk of being confronted with different ethical challenges became even more evident during the pandemic when care meeting the needs of older persons could not be provided.^9^ Older persons, particularly those living in RCFs, were highlighted as a group with a high risk of developing serious illness and death.^10^ According to the national COVID-19 Commission in Sweden, the high viral transmission in society, as well as structural deficiencies in residential care and lacking support to staff contributed to the situation in RCFs.^1^ In Sweden, as in other countries, visitor restrictions, cohort care and isolation were introduced, which affected residents’ physical and mental health^1,11^ and jeopardized their autonomy.^3^ In addition, older persons, especially those with dementia or other cognitive impairments, had difficulties understanding physical distancing or the need to use personal protective equipment (PPE), isolation and cohort care.^11^ A study has shown that, during the pandemic, persons with dementia suffered from social isolation and loneliness, which led to behavioural and psychological problems.^12^ Furthermore, studies of RCFs showed that residents experienced limited freedom, and loss of autonomy, social life and stimulation.^3^ Therefore, the pandemic had severe consequences for older persons living in RCFs.

Previous studies have shown that the differences in resources between hospitals and RCFs became very obvious, with there being a lack of medical assessments, treatment and PPE in the residential care environment.^11,12^ First-line managers were challenged in their leadership, as the prerequisites for care and the care environment changed rapidly throughout the pandemic.^13^ Studies have shown that staff were confronted with different ethical dilemmas during the pandemic because of the need to work with infected older persons; the dilemmas included compliance with PPE guidelines, isolating older persons by locking them in their rooms, staff who did not dare to leave the workplace, and fear of bringing COVID-19 infection to residents.^14^ During the pandemic, the workload of the staff therefore increased, which led to burnout, anxiety, insomnia and depression.^14^

The fundamental core of person-centredness is grounded in the rights and values of the unique person and based on ethical principles.^15,16^ Even if person-centred care is an established care model, it can be difficult to attain.^17^ During COVID-19 all the changes that occurred during the pandemic reduced the RCF residents’ autonomy, freedom, participation and wellbeing^3^ and increased the staff workload.^14^ However, little is known about leaders’ experiences of ethical challenges during this time.

## Aim

This study’s aim was to explore ethical challenges in relation to person-centredness during the COVID-19 pandemic, from the perspective of leaders in RCFs.

## Method

### Design

The study has a descriptive and exploratory design based on qualitative data with semi-structured interviews. It is a part of the PERLE project, involving Sweden and Australia, which has a focus on person-centred care and person-centred leadership in RCFs. The Consolidated Criteria for Reporting Qualitative Research (COREQ) checklist was followed.^18^

### Participants

The study used purposeful sampling. A total of 26 leaders, 12 RNs and 14 FLMs working in public or private RCFs in seven different municipalities in southern and northern Sweden, including rural and urban areas, participated in the study. Twenty-four interviews were conducted individually, while one interview was performed with two participants. Of the participants, four were men and 22 were women, aged between 30 and 65 years (Table 1). The inclusion criterion was having been a leader during the COVID-19 pandemic in an RCF. The heads of social services gave their permission to invite FLMs and RNs to participate in the study. Information about the study was provided at workplace meetings. The participants indicated their interest in participating directly to the first author.

**Table 1.** Characteristics of participants.

Characteristics	n = 26
Age, yrs, md	50.5
Range	30–65
Gender	
Woman/man	22/4
Working title	
Registered nurse/first-line manager	12/14
Qualification	
Registered nurse	15
Social worker	8
Other	3
Total working experience, yrs, md	9.5
Range	2–42
Education in person-centred care	
Yes/no	12/14
Leadership education	
Yes/no	11/15
Type of care facility^a^	
General residential care facility	17
Dementia care facility	14
Short-term accommodation	1
Number of residents at the facility^b^, md	27
Range	15–54
Number of personnel at the residential care facility, md	30
Range	14–80

^a^More than one alternative was possible.

^b^Two registered nurses (RNs) with night shift had responsibility for the entire residential care facility (RCF) (data not included).

### Study context

In Sweden, RCFs house persons ≥ 65 years who need around-the-clock care and services. Swedish municipalities are required by law to have RCFs, managed by public or private providers.^19^ The residents pay rent and fees for a home-like apartment of 30–40 m^2^. First-line managers have the overall responsibility for the RCFs, including residents, care staff such as enrolled nurses and nurse assistants and the work environment. The RNs are not subordinates of the FLM but are employed by a separate organization. The RNs have the main responsibility for the nursing care in the RCFs. This means that FLMs and RNs have a joint responsibility for the care practices. The residents receive personal care and social services from direct care staff.^19^

In Sweden there was no strict lockdown during the pandemic but visitor restrictions and social distancing, among other precautions, were applied.^1^ Restrictions were in place between March 2020 and March 2022. The vaccine was introduced in RCFs at the beginning of 2021. In January 2022, 95% of RCF residents had received vaccination against COVID-19.^20^

### Data collection

For the purposes of this study, semi-structured interviews were conducted.^21^ Data collection started in February 2022 and ended in March 2023. Three pilot interviews (RN and FLMs) were conducted prior to the data collection to test the interview guide. The interview guide (see Table 2) was thereafter slightly revised, and the pilot interviews were included in the results. Nineteen interviews were performed digitally, and six were conducted face to face at the participant’s workplace. All interviews were audio-recorded and lasted between 50 and 96 min (median 68 min). All interviews were conducted by the first author, except one that was conducted by the first and the last author together. The interviews were transcribed verbatim.

**Table 2.** Interview guide.

**Questions concerning ethical challenges**
Have there been any situations specifically linked to COVID-19?
How did you handle the situation and what consequences did it have?
Can you recall a situation when you had no influence on the circumstances?
Which values have been the most important?
Whose needs have been most important?
How did you experience…?
**Questions concerning person-centred care and ethical challenges**
The ethical challenges that you previously described, what significance did they have, and how did they affect the provision of person-centred care?
During the COVID-19 pandemic, were there any forums where you could discuss ethical challenges?^a^
What support have you received as a leader? Do you have any examples?^1^
What knowledge and support would you have liked?^a^

^a^not reported in this paper.

### Data analysis

A conventional content analysis was used.^22^ To obtain a sense of the whole, the interviews were read several times by all the authors. The next step was to derive codes relevant to the study aim^22^; to this end, the NVivo program^23^ was used. All codes were sorted into meaningful clusters (n = 22). Subcategories and categories were developed^22^ with a focus on differences and relationships between the clusters (Table 3). This resulted in four main categories with 13 subcategories representing internal variation. In the analysis, a main theme was formulated, which represents the overall understanding of the findings (Table 4).

**Table 3.** Examples from the analytical process.

Quotation	Code	Meaningful cluster	Subcategory	Category	Theme
It is an ethical dilemma whether to lock a resident in their room for 14 days, or to make them take the test and then let the residents have a nice life and socialize with others	Locking a resident up in their room versus making them take the test, whereafter they can socialize	Forced measures	Going against a resident’s will by performing a COVID test, or being forced to isolate them	Protecting the group versus promoting the older person’s autonomy	Having to disregard the individual needs of the person in order to protect the group and society
I couldn’t do anything – powerless. To not be able to relieve symptoms … There was much suffering for the resident. It took a long time for the prescription. I can’t do anything when I don’t have the right resources	Powerless to alleviate the suffering of the resident. Lacking the right resources to act	Inadequate resources	Being unable to provide care based on professional competence because of inadequate resources	Striving to provide dignified care but lacking opportunities

**Table 4.** Ethical challenges in residential care facilities during the COVID-19: leaders’ perspective.

Having to disregard the individual needs of the person in order to protect the group and society
• Protecting the group versus promoting the older person’s autonomy
- Having to apply visitor restrictions despite the residents’ need for social contact
- Prohibiting relatives from visiting even though the residents were at the end of their life
- Being forced to isolate the residents to avoid the spread of infection
- Maintaining distance despite the residents’ need for closeness and touch
- Going against a resident’s will by performing a COVID test, or being forced to isolate them
• Being forced to lead care based on uncertainty instead of evidence
- Struggling with an endless flow of information, but still being left with no answers
- Having to apply guidelines that go against one’s personal beliefs
- Standing alone in making decisions, and being held accountable
• Striving to provide dignified care but lacking opportunities
- Being unable to provide care based on professional competence because of inadequate medical resources
- Having to establish prioritization schemes for care, at the cost of individual needs
• Going far beyond ordinary duty and endangering one’s own and the staff’s health
- Being compelled to protect the staff, but still having to expose them to infection
- Feeling compelled to prioritize work over personal life
- Being forced to pressure staff to shoulder an unreasonable workload and risk crossing their limits

### Ethical considerations

The study was approved by the Swedish Ethical Review Authority (Dnr 2021–000413) and conducted in accordance with the Declaration of Helsinki.^24^ The participants received oral and written information about the study, and were assured that participation was voluntary, and that they could withdraw from the study at any time. Written consent was obtained. All personal data were coded and names and places were not included in the transcribed interviews to ensure confidentiality.

## Findings

The leaders’ main challenges during the pandemic were having to disregard the individual needs of the person in order to protect the group and society. This meant that instead of promoting person-centredness, they had to focus on the entire facility. It was ethically challenging to be forced to reduce personal interactions to protect themselves and others. It was difficult to ‘do good’ when trying to protect the residents from infections. This meant violating their integrity. Wearing PPE when having contact with the residents felt impersonal and was against the leaders’ own beliefs. The RCF became prison-like with all the guidelines and restrictions, which threatened the residents’ autonomy.

### Protecting the group versus promoting the older person’s autonomy

The leaders described ethical challenges in protecting the group versus promoting the resident’s autonomy. One such challenge was having to go against residents’ right to experience meaningfulness and engagement. Simultaneously, they had to consider what was best for all residents living there. Sometimes they had to decline residents’ wishes, based on concerns for the rest of the residents, and felt a sense of being torn apart as a result.

#### Having to apply visitor restrictions despite the residents’ need for social contact

Ethical challenges arose when visitor restrictions were introduced despite the residents’ need for social contact. The leaders perceived increased anxiety among the residents when they were prohibited from socializing with relatives and the other residents. Social activities were limited or cancelled altogether, including pedicures, hairdressing and shopping, as well as in-facility activities such as bingo, café and musical entertainment. *‘It became an ethical dilemma when the residents wanted a meaningful everyday life, but we couldn’t offer any activities’* (Interview 7). The leaders tried to encourage the staff to perform one-on-one activities with residents, but this could not fully compensate for the residents’ need for social interaction with others, and so the residents experienced loneliness and even depression.

#### Prohibiting relatives from visiting even though the residents were at the end of their life

The leaders said it was difficult to prohibit relatives from visiting the residents when the residents were at the end of their life. However, the alternative meant that both could be infected. Some relatives found it hard to accept that they were not allowed to visit; it did not matter to them if they became infected. The challenge for the leaders was that, on the one hand, it was difficult to estimate how much time the residents had left in life. *‘The visitor restrictions were ethically challenging. When you are old and live in an RCF, you don’t know if today is the last day of your life’* (Interview 6). On the other hand, the leaders did not want to risk having someone die alone, without relatives present. This was seen as unjustifiable care.

#### Being forced to isolate the residents to avoid the spread of infection

An ethical challenge was being forced to isolate the residents to avoid the spread of infection. While the leaders understood the importance of isolation, they perceived it as inhumane. It was particularly difficult to isolate residents with dementia; at the same time, it was not right to let one resident walk around the unit and infect other residents.

*Persons with dementia who don’t want to be in their rooms, well, it’s not a prison, we can’t force them. We can try to bring them back to their room, but if they don’t want to, we have no legal right to just lock them in*. (Interview 11)

Sometimes residents had to be isolated in their room for 14 days, sometimes longer, and it happened that residents were given sedative drugs to stay in their rooms, even though this was not considered ethically right. The leaders expressed that although isolation had a good purpose, it was ethically challenging to implement.

#### Maintaining distance despite the residents’ need for closeness and touch

An ethical challenge for the leaders was to maintain and ensure distance in spite of the residents’ need for closeness and touch. Closeness was effective in alleviate anxiety, but was not possible. To be distant when the residents wanted to be close was difficult and many leaders expressed a sense of being torn apart. *‘The residents seek contact all the time, and it’s very difficult to keep a distance. It becomes an internal conflict, and at the same time you have to acknowledge the residents’* (Interview 10). Sometimes the leaders chose to give a hug so that the residents would feel confirmed as human beings, even though it was not allowed. It was a challenge to use PPE; it created uncertainty in residents with limited communication. In end-of-life care, the staff were not even allowed to hold a resident’s hand without PPE. It was hard to ask relatives to wear full PPE when visiting a resident at end of life.

#### Going against a resident’s will by performing a COVID test, or being forced to isolate them

It was an ethical dilemma for the leaders to go against an older resident’s will and perform a COVID test or else isolate them. Even though it was sometimes possible to take a test, this was not always reliable because the resident waved the test swab away. Some residents refused a test, and they were then held under coercion. Sometimes sedative drugs were used to enable taking a COVID test. These situations were described as abusive. Even situations where residents were repeatedly persuaded to take a test felt like abuse, which had to be balanced against the importance of finding out whether someone was infected or not. Without the COVID test, a resident might have to be considered infected and be isolated for 14 days. Both outcomes were negative. *‘It is an ethical dilemma whether to lock a resident in a room for 14 days, or to make them take the test and then let the residents have a nice life and socialize with others’* (Interview 14).

### Being forced to lead care based on uncertainty instead of evidence

It was a challenge to lead care based on uncertainty instead of evidence. COVID-19 was unknown, it felt new and there was a lot of uncertainty. The situation was chaotic at the beginning and there was a sense of panic about how the disease should be handled. It was described as a war situation, with a feeling of judgement day. The leaders felt powerless. Doing what is ethically right and good was considerably more difficult in situations when there was a lack of knowledge to base their actions on.

#### Struggling with an endless flow of information, but still being left with no answers

A challenge was to deal with an endless flow of information. It was difficult to ‘do good and right’ while exposed to this high and intense flow of information. The information did not provide support and it constantly changed. The information about PPE, isolation and handling an outbreak of COVID-19 infection was ambiguous. This made it difficult to give the right information to staff and answer their questions. The leaders had to rely on what they thought was best and it felt ethically difficult with limited evidence to lean on. *‘How should we handle this situation? Sometimes there were no answers’* (Interview 15).

#### Having to apply guidelines that go against one’s personal beliefs

The leaders had to apply some guidelines that were against their own beliefs. The guidelines were not always reasonable; for example, being forced to conduct cohort care or apply visitor restrictions was perceived as unreasonable. It was deemed necessary to follow the guidelines and although these were against the leaders’ convictions, there had to be consistency. It was challenging to keep up with the requirements for cleaning and hygiene in the residents’ apartments and some leaders expressed how the RCF had changed from a home-like environment to an institution. This went against their beliefs with respect to offering the residents a home environment. *‘We are in their home. They live in an apartment, this is their home and here we come in with full PPE’* (Interview 3).

#### Standing alone in making decisions, and being held accountable

The leaders often felt alone in making decisions and were held accountable for them. One single decision could have severe consequences, such as the entire unit having an outbreak of COVID-19. Ethical situations arose when they had to consider whether to save themselves first or not.

*… feeling that I saved myself first, so I got a bad conscience. But if I stayed in the unit and talked, I got a bad conscience too: now I might infect someone there. It doesn’t matter how or what you do …* (Interview 1).

The leaders struggled with their conscience with regard to the decisions they had to take, for example, in end-of-life care. Other hard decisions were whether residents should remain at the RCF or be sent to hospital, as these decisions lay in their hands. Sometimes they had a colleague to discuss things with, but often they were alone in having to decide and they felt left on their own and responsible for the consequences of the decisions they took.

### Striving to provide dignified care but lacking opportunities

Another ethical challenge was the lack of opportunities to provide dignified care. The desire to provide dignified care was deeply rooted within the leaders. It was a challenge to dismiss something that felt right to do for the residents or to safeguard their dignity. The leaders explained that dignified care aims to provide opportunities to live a good life until the end at an RCF, without suffering.

#### Being unable to provide care based on professional competence because of inadequate resources

The leaders experienced ethical challenges when they were unable to provide care based on professional competence because of inadequate medical resources. They felt powerless when they were unable to use their competence and professional judgement. Without oxygen or other necessary resources, they could not relieve symptoms and perceived increased suffering among residents. *‘I couldn’t do anything – powerless. To not be able to relieve symptoms ... There was much suffering for the resident. It took a long time for the prescription. I can’t do anything when I don’t have the right resources’* (Interview 17). Medical examinations on site and consultations with physicians also failed. There was a lack of drugs in the hospitals and the leaders received orders that drugs were to be reserved for the hospitals, which gave the feeling that the residents in RCFs were ‘less valuable’.

#### Having to establish prioritization schemes for care, at the cost of individual needs

The leaders described that prioritization schemes were established for care, and this was perceived to be at the cost of the residents’ individual needs. With the prioritization schemes, it was not certain that residents would get help with showering, washing and cleaning and even with getting out of bed. *‘The basic needs, that you get food and medicine, that’s really it, but it happened that residents did not get up [from bed] because of the staff situation’* (Interview 7). As prioritization schemes were established, person-centred care decreased and, in some cases, ceased to exist at all. The care of the residents became more automated and the human element was reduced.

### Going far beyond ordinary duty and endangering one’s own and the staff’s health

The leaders described that they had to go far beyond ordinary duty and endanger their own and their staff’s health. They had to work under extreme conditions, and it was a challenge to determine how much the staff could be pushed without exceeding their health limits. The leaders put aside their fear of becoming infected when they dealt with a resident who had COVID-19, and they provided care regardless of what personal consequences it might have.

#### Being compelled to protect the staff, but still having to expose them to infection

An ethical challenge was to protect the staff but at the same time to expose them to the virus. Because of the lack of PPE early on, they sometimes had to ask staff to care for residents without wearing adequate PPE. *‘… when staff called and were anxious and I needed to tell them to care for this resident without enough PPE. This is not right, but at the same time we must take care of the residents’* (Interview 16). They even had to make visors of overhead paper, in contrast to the hospitals which had access to gas masks. It was ethically challenging when they had to send anxious and worried staff to infected persons, thereby putting their health and life at risk.

#### Feeling compelled to prioritize work over personal life

The leaders described that both they themselves and the staff gave up their private lives for work. The leaders expressed fear of bringing COVID home to their families and loved ones and for this reason avoided contact with them. They were also afraid of bringing COVID back to the RCF or spreading the infection between facilities. They were concerned about the residents all the time when they were at home or had a day off work. They were constantly available by phone round the clock during this period as it was against their conscience to ‘disconnect’ work during these circumstances. When the workload was very high, the leaders expressed that it was hard to manage everyday life at home, where they felt torn between living a private life and protecting society. *‘It was stressful, also in my private life. I remember one New Year’s Eve, making dinner, I called staff and said that you must work. I didn’t have the conscience to make myself unavailable’* (Interview 12).

#### Being forced to pressure staff to shoulder an unreasonable workload and risk crossing their limits

Another ethical challenge described by the leaders was being forced to pressure staff to shoulder an unreasonable workload and risk crossing their limits. *‘How much can I push the staff? How much can I ask from them and when do they need to rest?’* (Interview 2). Often the staff had to work long or double shifts or take on extra shifts at short notice. Over some periods, the staff were totally exhausted, and some reported sick. The staff sometimes offered to take on extra shifts, although they were already stretching their limits, which was ethically challenging for the leaders. The leaders were dependent on having staff to care for the residents, and there was no one else who could replace them.

## Discussion

The main finding was that leaders were forced to disregard the person's individual needs in order to protect the group and society. Maximizing what is good for most people is generally known as utilitarianism.^25^ The utilitarian approach facilitates quantitative aspects, such as the largest possible number of benefits.^26^ Therefore, the ethical challenges the leaders faced during the pandemic meant being torn between the two perspectives, with negative effects for the residents as individuals.

The restrictions prevented meeting the residents’ needs in a person-centred way. This happened when leaders had to protect the group rather than promoting an older person’s autonomy. The pandemic measures meant that the leaders had difficulty making the right decisions and ‘doing good’. Examples of these measures were visitor restrictions, isolating residents, COVID testing and maintaining distance, all of which deprived residents of autonomy. Nihlén Fahlqvist^27^ described that governments of different countries had to find the balance between protecting a group of people in society and ethical values and upholding fundamental rights for each individual during the pandemic. However, according to Samdal et al.,^28^ it was not ethically justifiable to isolate residents or ban visitors. Researchers have shown that the wellbeing of residents decreased as a result of pandemic measures.^29^ This highlights the consequences of being forced to maintain protective measures, endangering the group’s health through wanting to protect them. Also, the basis of person-centred care is promoting a good life for residents, relatives and staff, where all actions should support the individual’s autonomy and integrity.^30^ Furthermore, ethical principles are often deprioritized by other tasks,^30^ as became evident in the current study. The leaders faced ethical difficulties when it became impossible to practise person-centredness, and as a result ethical principles were violated such as respect for autonomy, and non-maleficence and beneficence. In agreement with our findings, Savage^13^ described that the clash between pandemic measures and person-centred values created inner conflict for leaders. Heggestad et al.^31^ showed that constrained person-centred practices negatively impact dignity and care quality. Furthermore, not being able to provide person-centred care has been related to more job strain^32^ and more stress of conscience^33^ among staff. This has implications for promoting person-centredness for both staff and residents.

The leaders had to handle a large amount of information, which was not always efficacious, with sometimes limited transferability to practice. Often, they had to lead the care based on uncertainty instead of evidence. Dixon et al.^34^ showed that potential risks related to residents’ wellbeing were missing in guidelines from authorities, as these were not prioritized. According to Hillestad et al.,^35^ one main ethical dilemma for leaders was to bear the responsibility on their own and manage the daily care of the residents on their own. This was also evident in the current study. For leaders, this meant that they had to solve problems on their own without evidence to guide them, and this negatively affected the quality of care.

Furthermore, the leaders strived to provide dignified care but lacked opportunities to do so. Not being able to provide adequate care is known to increase moral distress. Jameton^36^ defined moral distress as a phenomenon in which one knows the right action, but cannot pursue it owing to institutional constraints. Moral distress is similar to the concept of stress of conscience as both can be generated when organizational constraints prevent staff from providing the care they want to give. This is, however, not the only reason for stress of conscience, which term refers to the stress of a troubled conscience regardless of its cause.^37^ As described by Simonovich et al.,^38^ the major emotional response to providing care during the pandemic was moral distress, including feelings of fear, frustration, powerlessness and guilt. Moral distress in the care of residents has, moreover, been shown to result in increased sick leave as well as in staff leaving their jobs.^39^ However, already prior to the pandemic, staff working in the municipality experienced a heavy workload.^40,41^ The findings further showed that leaders had unreasonable demands and were forced to expose staff to life-threatening risks in terms of workload and infection exposure. It meant going far beyond duty and putting their own and their staff’s health at risk. The ethical challenges leaders and staff experienced will have long-lasting consequences with a potential risk of increased staff turnover. This has implications for stakeholders and management, who need to address and prioritize the work conditions and workload in these environments.

### Methodological considerations

Data were collected 2–3 years after the first outbreak of COVID-19 in Sweden, which may have led to recall bias. However, the participants gave detailed descriptions and had time to reflect on their answers.^42^ The results are consistent with other studies, which strengthens the credibility of the study. Other strengths of this study are that the participants represented different geographical areas in Sweden, on the basis that the spread of infection in areas in Sweden varied; this increased the transferability.

## Conclusion

The ethical challenges the leaders experienced during the pandemic meant being torn between two conflicting liabilities: meeting the person’s individual needs versus protecting the group and society. The leaders faced several ethically difficult situations resulting in undignified and compromised person-centred care. This has implications for stakeholders and management, who need to address and prioritize the work conditions and workload in these environments.
